# Systematic Identification of Host Immune Key Factors Influencing Viral Infection in PBL of ALV-J Infected SPF Chicken

**DOI:** 10.3390/v12010114

**Published:** 2020-01-16

**Authors:** Manman Dai, Shibing Li, Keyi Shi, Jiayu Liao, Hui Sun, Ming Liao

**Affiliations:** 1 College of Veterinary Medicine, South China Agricultural University, Guangzhou 510642, China; lishibin10086@163.com (S.L.); huanongshiky@163.com (K.S.); ljy153034045@163.com (J.L.); swhsunhui@163.com (H.S.); 2 National and Regional Joint Engineering Laboratory for Medicament of Zoonosis Prevention and Control, Guangzhou 510642, China

**Keywords:** chicken, ALV-J, CD8^+^ T cell response, phenotype, PBL

## Abstract

Although research related to avian leukosis virus subgroup J (ALV-J) has lasted for more than a century, the systematic identification of host immune key factors against ALV-J infection has not been reported. In this study, we establish an infection model in which four-week-old SPF chickens are infected with ALV-J strain CHN06, after which the host immune response is detected. We found that the expression of two antiviral interferon-stimulated genes (ISGs) (Mx1 and IFIT5) were increased in ALV-J infected peripheral blood lymphocytes (PBL). A significant CD8^+^ T cell response induced by ALV-J appeared as early as seven days post-infection (DPI), and humoral immunity starting from 21 DPI differed greatly in the time scale of induction level. Meanwhile, the ALV-J viremia was significantly decreased before antibody production at 14 DPI, and eliminated at 21 DPI under a very low antibody level. The up-regulated CD8^+^ T cell in the thymus (14DPI) and PBL (7 DPI and 21 DPI) was detected, indicating that the thymus may provide the output of CD8^+^ T cell to PBL, which was related to virus clearance. Besides, up-regulated chemokine CXCLi1 at 7 DPI in PBL was observed, which may be related to the migration of the CD8^+^ T cell from the thymus to PBL. More importantly, the CD8 ^high+^ T cell response of the CD8αβ phenotype may produce granzyme K, NK lysin, or IFN-γ for clearing viruses. These findings provide novel insights and direction for developing effective ALV-J vaccines.

## 1. Introduction

The avian leukosis virus subgroup J (ALV-J) has caused severe economic losses in the poultry industry worldwide due to its great pathogenicity and transmission ability [1]. Unfortunately, there are still no vaccines or drugs which effectively protect against ALV-J infection. Thus, the big project, ALV eradication, has become a major measure for the prevention and control of ALV-J, which is funds-, manpower-, and technology-dependent. Besides, factors such as the fluctuation of reproductive hormones would also influence the efficiency of eradication via inducing ALV-J viremia disappearance in positively infected chickens [2]. Not surprisingly, ALV eradication is a big challenge, especially in developing countries like China. Therefore, some researchers are still devoted to developing various vaccines, such as subunit vaccines, by using the gp85 protein of ALV-J, multi-epitope subunit vaccine, and chimeric multi-epitope-based DNA vaccine [3,4,5,6]. However, none of these vaccines are commercial or applied in chicken farms. In fact, a full understanding of the ALV-induced adaptive immune response is the premise to developing effective vaccines. Regretfully, to our knowledge, the systematic identification of host immune key factors against ALV-J infection has not been reported, and still less the immunodominant viral antigen or epitope eliciting the effective immune response.

ALV-J as a kind of notorious retrovirus could cause neoplastic disease, immunosuppression, and other production problems. Historically, ALV studies have mainly focused on the virus itself, including exploring the mechanisms of tumorigenesis, virus isolation, viral replication, and pathogenesis [7,8,9]. However, ALV-related immunology research is still in its infancy. Despite our knowledge of immunosuppression, immunologic tolerance, antibody response, and interferon-stimulated gene (ISG) expression induced by ALV-J [10,11,12,13], there are still major gaps in our understanding of immune responses against ALV-J infection. In a previous study, an infection model was established in which seven- or 11-day-old embryos, and one- or seven-day-old SPF chickens were infected with the virus and used to study the pathogenesis of ALV-J, including tumorigenesis, immunosuppression, and immunologic tolerance [9,10,11]. In this study, four-week-old, fully developed SPF chickens with immune organs were used to study the host optimal immune response after ALV-J infection. This infectious model was also able to be a reference for studying other virus-induced optimal immune responses.

Previously, we summarized the progress on chicken T cell immunity to viruses, and found that no ALV-related T cell epitope was functionally identified [14], implying the present scarcity in the study of the ALV-J induced cell immune response. Here, we systematically identified the host immune response in peripheral blood lymphocytes (PBL) of ALV-J infected SPF chickens and elucidated the potential key factors influencing viral infection, especially the phenotype and function of the important T cell response.

## 2. Materials and Methods

### 2.1. Ethics Statement

All animal research projects were sanctioned by the South China Agriculture University Institutional Animal Care and Use Committee (identification code: 2019076, 10 June 2019). All animal procedures were performed according to the regulations and guidelines established by this committee and international standards for animal welfare.

### 2.2. Virus and Experimental Animals

The ALV J subgroup strain CHN06 was isolated and identified by our laboratory [9]. A total of 28 two-week-old, specific-pathogen-free (SPF) White Leghorn chickens (Guangdong Da Hua Nong Animal Health Products Co., Ltd., Guangdong, China) were randomly assigned to two groups, namely, a CHN06-infected group and control group, each with 14 chickens per group which were reared separately in negative-pressure isolators. After feeding for two weeks, four-week-old SPF chickens were inoculated intraperitoneally at a dose of 0.8 mL (10^4^ TCID_50_/0.1 mL) of strain CHN06. The control group was injected with 0.8 mL PBS alone. At 14 and 28 days post-infection (DPI), two infected chickens and three control chickens were humanely sacrificed. At 42 DPI, three chickens per group were humanely euthanized. The spleens and thymus were aseptically collected at each time point, respectively.

### 2.3. ALV-J Shedding, ALV-J Viremia, ALV-J Antibody Detection

To monitor virus shedding, cloacal swabs were collected from chickens from 7 DPI to 63 DPI, and preserved in the diluent of ALV-P27 Ag Test Kit (IDEXX, Inc., Westbrook, MA, USA). Levels of p27 expression in cloacal swabs were examined following the manufacturer’s instructions. To monitor viremia, plasma samples were aseptically collected from chickens from 7 DPI to 63 DPI and centrifuged at 4 °C at 2000 rpm for 12 min to isolate leukocytes. These were then inoculated into DF1 cells at 37 °C for seven days to check for the presence of the virus by using an ALV-P27 Ag Test Kit (IDEXX, Inc.). The relative antigen titer level was determined by calculating the S/P ratio, and the cloacal swabs and plasma samples with a S/P ratio higher than 0.2 were considered virus-positive.

To monitor the anti-ALV-J antibody titers, the serum samples were collected from chickens from 7 DPI to 63 DPI and examined for the level of ALV-J antibody using a commercial ALV-J antibody test kit (IDEXX, Inc.) according to the manufacturer’s protocol. The relative antibody level in the serum was determined by calculating the S/P ratio, and a ratio higher than 0.6 was considered ALV-J antibody-positive.

### 2.4. Lymphocyte Isolation

Five days before infection (DBI) and each week after infection, heparinized blood samples from individual chickens were collected to isolate peripheral blood lymphocytes (PBL) of chicken lymphocyte separation medium according to the manufacturer’s instructions (Solarbio, Beijing, China). Briefly, the heparinized blood was slowly added to the top of the lymphocyte separation medium and then centrifuged with 400 g for 15 min when the falling acceleration was set to 1. After centrifugation, three layers appeared, and the interlayer was lymphocyte. The interlayer was transferred to a new tube and washed with cleaning fluid. After centrifugation with 1700 rpm for 5 min, red-blood-cell lysis buffer (Haoyang, Tianjin, China) was used to remove the mixed blood cells. After that, the PBL was prepared.

Single-cell suspensions derived from the spleen and thymus were obtained in RPMI 1640 medium (Gibco, CA, USA) of the tissue mononuclear cell kit, according to the manufacturer’s instructions (Haoyang, Tianjin, China). Briefly, tissue cell suspensions were obtained by mechanical disruption. The lymphocyte was isolated with the tissue separation medium in the kit, as described above. Cell viability and counting was performed using Trypan Blue and a Neubauer hemocytometer (Sigma-Aldrich, St. Louis, MO USA). The chicken PBL and tissue single-cell suspensions were frozen in liquid nitrogen for later study.

### 2.5. Flow Cytometry

3 × 10^5^ cells of PBL or tissue single-cell suspensions were simultaneously incubated with APC-conjugated mouse anti-chicken CD3^+^, FITC-conjugated mouse anti-chicken CD4^+^, and PE-conjugated mouse anti-chicken CD8α^+^ monoclonal antibodies (SouthernBiotech, Birmingham, USA) in the dark at 4 °C for 30 min. After three washes with PBS, the labeled cells were analyzed by flow cytometer (CytoFLEX, Beckman Coulter, Brea, CA, USA) within 12 h. The data were analyzed by the software of FlowJo V10 (TreestarInc, Ashland, OR, USA). For the phenotype identification of the CD8^+^ T cell, FITC-conjugated mouse anti-chicken CD8β^+^ and APC-conjugated mouse anti-chicken CD4^+^ monoclonal antibodies (SouthernBiotech, Birmingham, USA) were also used.

### 2.6. Expression Analysis of Immune-Related Gene by qRT-PCR

Expressions of the immune-related gene were analyzed by a quantitative real-time polymerase chain reaction (qRT-PCR). Total RNA was extracted from 4 × 10^6^ live cells of post-thawed PBL using the RNAfast200 kit (Fastagen, Shanghai, China), followed by cDNA synthesis of mRNA with the RevertAid First strand cDNA synthesis kit (Thermo-Fisher Scientific, Shanghai, China) according to the manufacturer’s instructions. qRT-PCR was performed on an ABI7500 Real-Time PCR system (Applied Biosystems, USA) using iTaqTM Universal SYBR R Green Supermix Kit reagents (Biorad, CA, USA) according to the manufacturer’s specifications. Primers used for qRT-PCR were listed in Table 1. Data analyses were performed using the 2^−∆∆*C*t^ method [15].

### 2.7. Statistical Analyses

Statistical comparisons were made by GraphPad Prism 5 (GraphPad Software Inc., San Diego, CA, USA). The results were presented as mean ± SEM. * *p* < 0.05, ** *p* < 0.01, *** *p* < 0.001, ns indicates not significant.

## 3. Results

### 3.1. Dynamic Detection of ALV-J Shedding, ALV-J Viremia, and ALV-J Antibody

**Figure 1:**
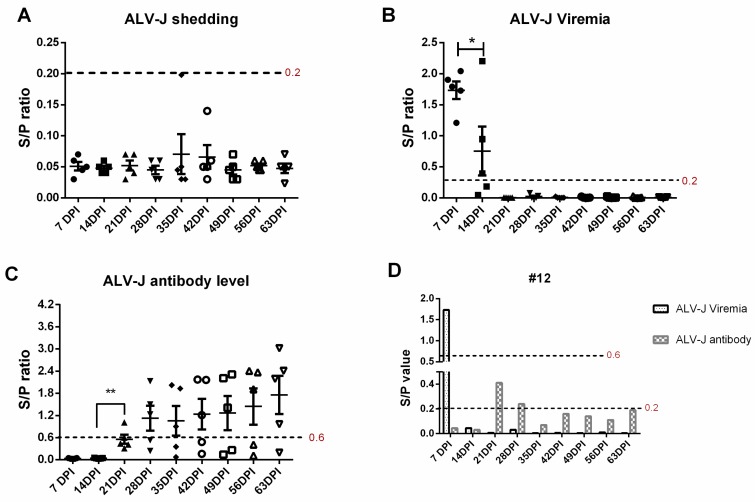
Dynamic detection of the avian leukosis virus subgroup J (ALV-J) shedding, ALV-J viremia, and ALV-J antibody. Five chickens were randomly selected for sampling every seven days post-infection (DPI). (**A**) ALV-J shedding was monitored via detecting the p27 expression levels in cloacal swabs. S/P value below 0.2 (red) indicated negative ALV-J shedding. (**B**) ALV-J viremia was monitored via virus isolation with the routine method. An S/P value above 0.2 (red) indicated positive ALV-J viremia. (**C**) The ALV-J antibody level in the serum was monitored using the commercial ALV-J antibody test kit. An S/P value above 0.6 (red) was considered ALV-J antibody positive. (**D**) ALV-J viremia and antibody level of chicken number 12 were analyzed from 7 DPI to 63 DPI. The paired *t*-test was used for statistical comparison. * *p* < 0.05, ** p < 0.01.

Four-week-old SPF chickens were infected with ALV-J for 63 days, and the ALV-J shedding, ALV-J viremia, and ALV-J antibody were continuously monitored every seven days. In the control group, the ALV-J shedding, ALV-J viremia, and ALV-J antibody are all negative at all time points. In the ALV-J infected group, ALV-J shedding detection by the cloacal swab-specific p27 antigen is negative with a S/P ratio below 0.2 at all the time points (Figure 1A). Besides this, ALV-J viremia detection by plasma sample-specific p27 antigen is 100% positive at 7 DPI, 60% positive at 14 DPI, and 100% negative at later time points. Moreover, the viral load of plasma samples is much lower at 14 DPI than 7 DPI (*p* < 0.05, Figure 1B). Additionally, the ALV-J antibody is 100% negative at 7 DPI and 14 DPI, 20% positive at 21 DPI, 60% positive from 28 DPI to 56 DPI, and 80% positive at 63 DPI. Also, the average ALV-J antibody level is slightly growing at a gradual rate (Figure 1C). Intriguingly, ALV-J infection is significantly suppressed at 14 DPI without antibody production (Figure 1B,C), which indicates that other host immune factors play a crucial role against ALV-J infection. More importantly, we found that ALV-J infection had been eliminated in four infected chickens without antibody production (Table S1). As shown in Figure 1D, the viremia in infected chicken number 12 disappeared at 14 DPI without antibody production in the whole monitoring process, which implies that the antibody—namely, humoral immunity—may not be the key or necessary factor to defending against ALV-J infection.

### 3.2. Dynamic Detection of T Lymphocyte Percentage

**Figure 2:**
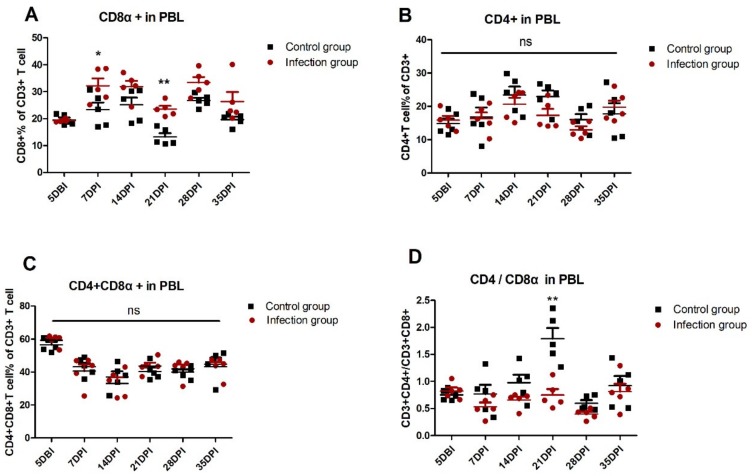
Dynamic detection of T lymphocyte percentage in peripheral blood lymphocytes (PBL). Five days before infection (DBI) and each week after infection, PBL derived from five chickens of infected and control groups were isolated to detect the T lymphocyte percentage, including the percentage of (**A**) the CD3^+^CD8^+^ T cell, (**B**) CD3^+^CD4^+^ T cell, and (**C**) CD3^+^CD4^+^CD8^+^. (**D**) The ratio of CD3^+^CD4^+^/CD3^+^CD8^+^ was detected. Each sample collected 1 × 10^5^ cells for flow cytometric analysis. The two-way ANOVA was used for statistical comparison. ns *p* > 0.05, * *p* < 0.05, ** *p* < 0.01.

**Figure 3:**
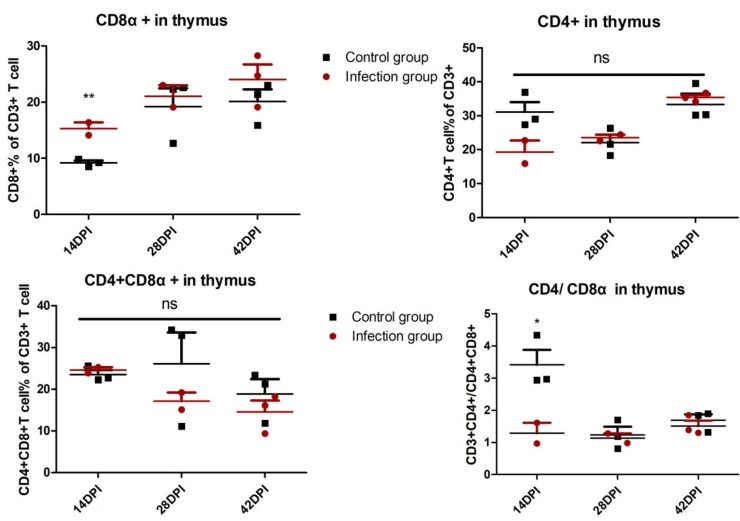
Analysis of T lymphocyte percentage in the thymus. Thymus single-cell suspensions derived from chickens of infected and control groups were isolated to detect the T lymphocyte percentage. Every dot stands for one chicken. The unpaired t-test was used for statistical comparison. ns *p* > 0.05, * *p* < 0.05, ** *p* < 0.01. Each sample collected 2 × 10^5^ cells for flow cytometric analysis.

**Figure 4:**
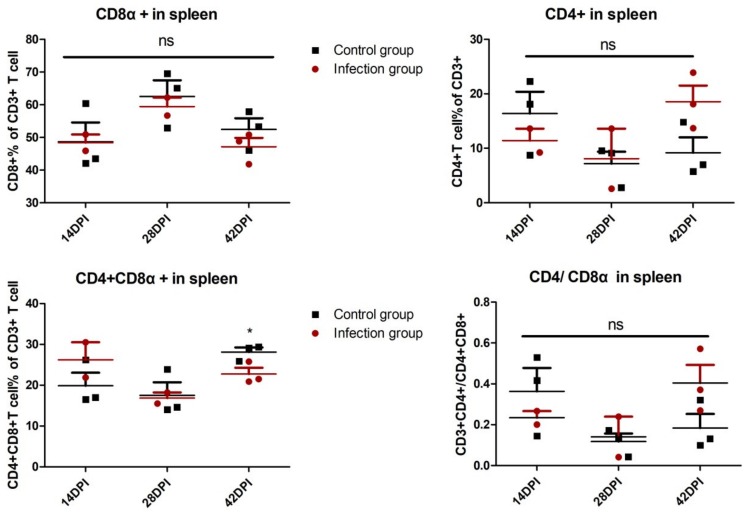
Analysis of T lymphocyte percentage in spleen. Spleen single-cell suspensions derived from chickens of infected and control groups were isolated to detect the T lymphocyte percentage. Every dot stands for one chicken. The unpaired t-test was used for statistical comparison. ns *p* > 0.05, * *p* < 0.05.. Each sample collected 2 × 10^5^ cells for flow cytometric analysis.

In order to explore the role of cellular immunity against ALV-J infection, the T lymphocyte percentage was detected in PBL and immune organs. As shown in Figure 2, in PBL, the percentage of CD8^+^ T lymphocytes in the infection group obviously increased at 7 DPI *(p <* 0.05) and 21 DPI (*p* < 0.01) compared with the control group. On the contrary, there is no significant difference about the percentage of CD4^+^ T cells and CD4^+^ CD8^+^ double-positive T cells in PBL between the infection group and control group. The ratios of CD4^+^/CD8^+^ in PBL of the infected group are markedly lower than the control group at 21 DPI. Similarly, in the thymus, the percentage of CD8^+^ T lymphocytes and the ratios of CD4^+^/CD8^+^ in the infection group is significantly up- and down-regulated, respectively, at 14 DPI (*p* < 0.01 and *p* < 0.05) compared with the control group (Figure S1A, Figure 3). In the spleen, the percentage of CD4^+^ CD8^+^ double-positive T cells in the infection group declined at 42 DPI (*p* < 0.05) compared with the control group, but there is no obvious difference at the other test index (Figure S1B, Figure 4). The fully-developed naïve T cell in the thymus could be located in the peripheral lymphoid organ via blood circulation [24]. In this study, we detected the up-regulated CD8^+^ T cell in the thymus (14 DPI) and PBL (7 DPI and 21 DPI), indicating that the thymus may provide the output of the CD8^+^ T cell to PBL, which was related to virus clearance.

### 3.3. Kinetic Detection of Immune-related Genes in PBL

**Figure 5:**
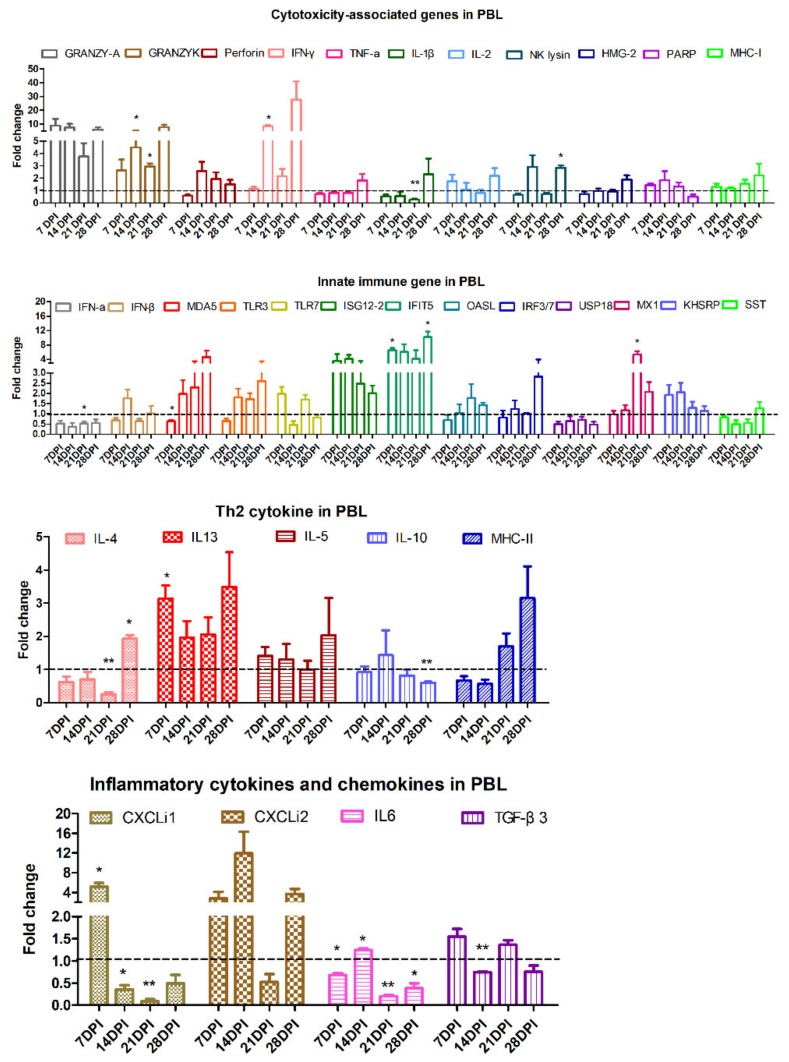
Analysis expression of immune-related genes in PBL by qRT-PCR. Expressions of immune-related genes in PBL were detected by qRT-PCR. The total RNA of PBL was extracted from three chickens of the infected and control groups, respectively. The data was collected from three biological samples in each group, each sample performed in triplicate. The results were presented as means ± SEM and the paired *t*-test was used for statistical comparison. ns *p* > 0.05, * *p* < 0.05, ** *p* < 0.01.

The kinetic expressions of immune-related genes at various time points were detected in PBL after ALV-J infection (Figure 5). Specifically, the transcriptional expression level of cytotoxicity genes, including granzyme K at 14 DPI and 21 DPI, interferon-γ (IFN-γ) at 14 DPI, and NK lysin at 28 DPI, and T helper (Th) 2 cytokines, including interleukin-13 (IL-13) at 7 DPI and IL-4 at 28 DPI, and ISG genes, including interferon-induced proteins with tetratricopeptide repeats (IFIT) 5 at 7 DPI and 28 DPI, myxovirus resistance 1 (MX1) at 21 DPI, inflammatory cytokines IL-6 at 14 DPI, and chemokines CXCLi1 at 7 DPI were significantly upregulated *(p <* 0.05) after ALV-J infection.

### 3.4. CD8^+^ T Cell Phenotype Identification

**Figure 6:**
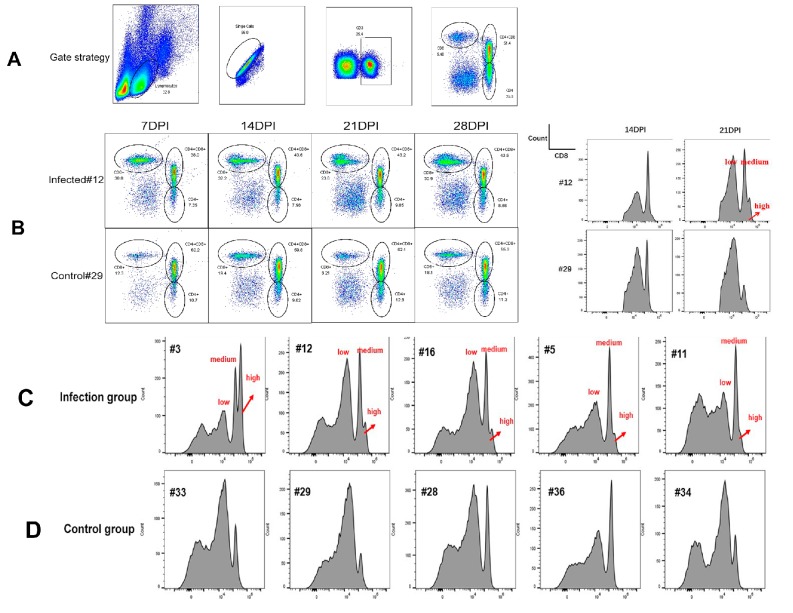
T cell phenotype analysis. (**A**) Gating of CD8α^+^, CD4^+^, and CD4^+^CD8α^+^ T cells with the CD3^+^(APC), CD4^+^(FITC), and CD8α^+^(PE) antibodies. (**B**) Analysis of the percentage and phenotype of T cells in PBL of ALV-J infected chicken number 12 and control chicken number 29 at various time points. Histogram of the CD3^+^CD8α^+^ T cells in PBL of (**C**) ALV-J infected group and (**D**) control group at 21 DPI. Each sample collected 1 × 10^5^ cells for flow cytometric analysis.

**Figure 7:**
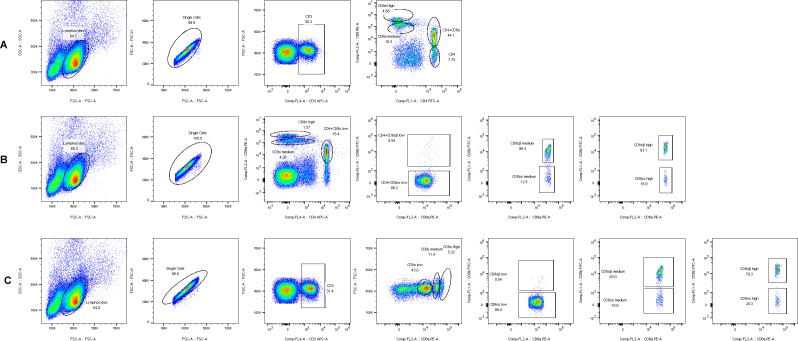
CD8^+^ T cell phenotype identification in PBL of ALV-J infected chicken number 12. (**A**) Gating strategy for analysis of CD8^high^α^+^, CD8^medium^α^+^, CD4^+^, and CD4^+^CD8^low^α^+^ T cells with the CD3^+^(APC), CD4^+^(FITC), and CD8α^+^(PE) antibodies. (**B**) Gating strategy for analysis of CD8^+^ αα and CD8^+^αβ phenotype in three CD8^+^ populations with the CD4^+^ (APC), CD8β^+^ (FITC), and CD8α^+^ (PE) antibodies. (**C**) Gating strategy for analysis of CD8^+^ αα and CD8^+^αβ phenotype in three CD8^+^ populations with the CD3^+^ (APC), CD8β^+^ (FITC), and CD8α^+^ (PE) antibodies. Each dot indicated each collected cell. And the circle or fame indicated the gated target cell population labelled by various antibodies. Each sample collected 1 × 10^5^ cells for flow cytometric analysis.

**Figure 8:**
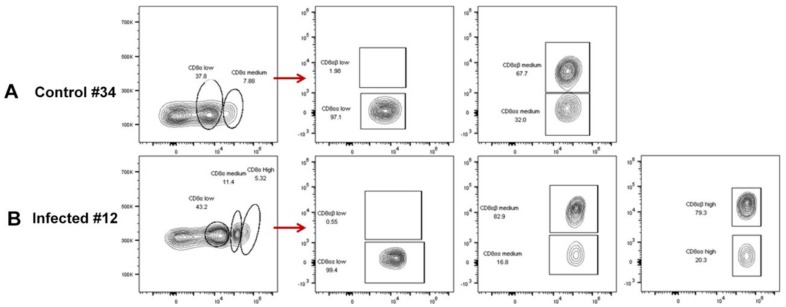
CD8^+^ T cell phenotype analysis. Contour plot of CD8^+^ αα and CD8^+^αβ T cells in PBL of (**A**) control chicken number 34 and (**B**) ALV-J infected chicken number 12 with the CD3^+^ (APC), CD8β^+^ (FITC), and CD8α^+^ (PE) antibodies. Each sample collected 1 × 10^5^ cells for flow cytometric analysis. The circle or fame indicated the gated target cell population labelled by various antibodies. And the red arrow indicated that each CD8α^+^T cell population was further subdivided to CD8αα and CD8αβ phenotype.

As described above, the increased percentage of CD8^+^ T lymphocytes in the PBL of the infection group was detected. More interestingly, we found that, compared with uninfected chicken, a CD8 ^high+^ population had appeared in the ALV-J infected chicken from 21 DPI, which then formed three stable populations of CD8^+^ T lymphocytes in infected chickens, including CD8 ^high+^, CD8 ^medium+^, and CD4^+^CD8 ^low+^ (Figure 6). Besides, the CD8^+^ T lymphocyte included two phenotypes, CD8αα and CD8αβ, and CD8αβ was expressed on normal cytotoxic T cells [25]. Thus, we further investigated the phenotype of CD8^+^T cells in ALV-J infected chicken number 12 via three combinations of antibody staining. The results showed that almost all of the CD4^+^CD8 ^low+^ population were of the CD4^+^CD8αα phenotype (Figure 7B). Conversely, the majority of the CD8 ^high+^ and CD8 ^medium+^ population was of the CD8αβ phenotype (Figure 7B,C). The contour plot analysis of the CD8^+^T cell phenotype in the PBL of control chicken number 34 and ALV-J infected chicken number 12 further confirmed the above results (Figure 8). Accordingly, these results implied that the CD8 ^high+^ population with the CD8αβ phenotype seemed to play a vital role in virus clearance.

## 4. Discussion

As an avian retrovirus, ALV-J can integrate into the host genome, causing tumor disease and inducing immunological tolerance mainly via the vertical transmission, which makes it very hard to eliminate and control. So far, ALV-J eradication has been the main strategy used to control it via eliminating positively infected chickens. Although it works, it does not mean that it has been defeated, since little is known about the host’s immune response to the virus. Previously, our research team and other researchers performed numerous studies on the innate immune response to ALV-J infection, as well as the interaction between ALV-J and macrophage, monocyte, and dendritic cells [12,13,17,26,27]. However, studies on the cellular immune response to ALV-J infection and the interaction between ALV-J and T cell are very few. More importantly, no study has been conducted about the systematical identification of host immune key factors against viral infection.

In this study, we established an infection model in which four-week-old SPF chickens were infected with ALV-J, after which the optimal host immune response was detected. We found that the CD8^+^ T cell response in PBL was obviously triggered after ALV-J infection at as early as 7 DPI (*p* < 0.05) and 21 DPI (*p* < 0.01) (Figure 2A). The production of the ALV-J antibody started from 21 DPI, which then slightly increased (Figure 1C). These results imply that ALV-J infection induced a cellular immune response, and the humoral immunity differed greatly in the time scale of the induction level, with a clearly detectable hierarchy. The ALV-J viremia was significantly decreased before antibody production at 14 DPI (Figure 1B,C). More interestingly, ALV-J viremia of four chickens could even be eliminated without antibody production (Figure 1C,D, Table S1), which suggests that the CD8^+^ T cell response was the potential key factor to defending against ALV-J infection. The thymus could provide the naïve T cell to the peripheral lymphatic organs via blood circulation [24]. Therefore, the obvious up-regulated percentage of CD8^+^ T cells in the thymus at 14 DPI (*p* < 0.01) may indicate the continuous output of CD8^+^ T cells from the thymus to the PBL. Studies have shown that the lower ration of CD4^+^/CD8^+^ could induce immune suppression [28,29]. Here, we also found that the ration of CD4^+^/CD8^+^ in the thymus at 14 DPI (*p* < 0.05) and the PBL at 21 DPI (*p* < 0.01) decreased after ALV-J infection, which implied that the virus may have exerted the immunosuppressive effect at this period.

Immune-related genes are important mediators and regulators of the host immune response against foreign antigens. For detecting the expression profile of immune-related genes in the PBL after ALV-J infection, we picked up four kinds of immune-related genes, including the cytotoxicity gene, Th2 cytokine, innate immune gene, and inflammatory cytokines and chemokines to analyze them according to the published papers, as shown in the reference of Table 1. Previously, the transcriptional expression level of cytotoxicity-associated genes were investigated in chickens infected with Marek’s Disease Virus (MDV), the infectious bursal disease virus (IBDV), and avian infectious bronchitis virus (IBV) [23,30,31,32]. In this study, we found that granzyme K, NK lysin, and IFN-γ were up-regulated (*p* < 0.05), which may be associated with the stage of activation of cytotoxic T lymphocytes (CTLs) in ALV-J infected PBL. Besides, the Th2 cytokines, including IL-13 and IL-4, were increased (*p* < 0.05), which may have been involved in assisting the humoral immune response [18,33]. ISGs have been found to be critical for controlling virus infections [34]. Here, we found that ALV-J infection triggered the up-regulated expression of two chicken antiviral ISGs, Mx1 [35] and IFIT5 [36]. Chemokine could regulate the circulation of immune cells and their recruitment to sites of infection. The mRNA expression of CXCLi1 and CXCLi2 can be induced in lymphocytes, heterophils, and oviduct epithelial cells [37,38]. We found that CXCLi1 at 7 DPI was obviously increased in ALV-J infected PBL (*p* < 0.05), which may be related to the migration of the CD8^+^ T cell from the thymus to PBL.

The above results indicated that the CD8^+^ T cell response, accompanied by the cytotoxicity-associated genes, including granzyme K, NK lysin, and IFN-γ, may have been the key factor to defending against ALV-J infection in PBL. Given these findings, we further identified the phenotype of the CD8^+^ T cell response after ALV-J infection. The experiments presented in the current study showed that a CD8 ^high+^ population appeared in the ALV-J infected chicken from 21 DPI compared with uninfected chicken (Figure 6). It is well-known that CD8 is expressed either as an αα-homodimer or αβ-heterodimer, and CD8αβ is expressed in normal cytotoxic T cells [25]. In this study, we found that the majority of the CD8 ^high+^ population were of the CD8αβ phenotype (Figure 7B,C and Figure 8). Accordingly, the CD8 ^high+^ population with a CD8αβ phenotype seemed to play a vital role in virus clearance. The specific effect response mechanism of this CD8 ^high+^ population needs to be further verified. For example, identifying immunodominant viral antigens or epitopes eliciting this effective immune response would guide the development and design of effective ALV-J vaccines.

In summary, for the first time, this study systematically explored host immune key factors against viral infection in the PBL of ALV-J infected SPF chickens. We found that the CD8 ^high+^ T cell response of the CD8αβ phenotype may produce granzyme K, NK lysin, or IFN-γ for clearing the virus. These findings provide novel insights and directions for developing effective ALV-J vaccines.

## Figures and Tables

**Table 1:** Primers used for the quantitative real-time polymerase chain reaction (qRT-PCR).

Target	Primer	Sequence (5′–3′)	Gen Bank Accession No.	Reference
GAPDH	Forward	GAACATCATCCCAGCGTCCA	NM_204305.1	[16]
	Reverse	CGGCAGGTCAGGTCAACAAC		
IFN-α	Forward	GACAGCCAACGCCAAAGC	GU119896.1	[16]
	Reverse	GTCGCTGCTGTCCAAGCATT		
IFN-β	Forward	GCCCACACACTCCAAAACACTG	NM_001024836.1	[16]
	Reverse	TTGATGCTGAGGTGAGCGTTG		
IL-1β	Forward	GGTCAACATCGCCACCTACA	NM_204524.1	[16]
	Reverse	CATACGAGATGGAAACCAGCAA		
TNF-α	Forward	GCTGTTCTATGACCGCCCAGTT	NM_204267.1	[16]
	Reverse	AACAACCAGCTATGCACCCCA		
IL-2	Forward	GCTAATGACTACAGCTTATGGAGCA	AF000631.1	[16]
	Reverse	TGGGTCTCAGTTGGTGTGTAGAG		
IL-6	Forward	AAATCCCTCCTCGCCAATCT	AJ309540.1	[16]
	Reverse	CCCTCACGGTCTTCTCCATAAA		
IL-10	Forward	AGCAGATCAAGGAGACGTTC	NM_001004414.2	[16]
	Reverse	ATCAGCAGGTACTCCTCGAT		
TLR3	Forward	ACAATGGCAGATTGTAGTCACCT	NM_001011691.3	[16]
	Reverse	GCACAATCCTGGTTTCAGTTTAG		
TLR7	Forward	TCTGGACTTCTCTAACAACA	NM_001011688.2	[16]
	Reverse	AATCTCATTCTCATTCATCATCA		
MHC-I	Forward	AAGAAGGGGAAGGGCTACAA	NM_001031338.1	[16]
	Reverse	AAGCAGTGCAGGCAAAGAAT		
MHC-II	Forward	CTCGAGGTCATGATCAGCAA	DQ008588.2	[16]
	Reverse	TGTAAACGTCTCCCCTTTGG		
IFN-γ	Forward	CCTCCAACACCTCTTCAACATG	X92479	[17]
	Reverse	TGGCGTGCGGTCAAT		
IL-4	Forward	TCGAGGAGTGACGGGTG	AJ621249.1	[17]
	Reverse	ACTATCCGGATGCTCTCCATC		
IL-5	Forward	GGAACGGCACTGTTGAAAAATAA	AJ621252.1	[18]
	Reverse	TTCTCCCTCTCCTGTCAGTTGTG		
IL-13	Forward	CTGCCCTTGCTCTCCTCTGT	AJ621250.1	[18]
	Reverse	CCTGCACTCCTCTGTTGAGCTT		
Granzyme A	Forward	ACTCATGTCGAGGGGATTCA	NM_204457.1	[19]
	Reverse	TGTAGACACCAGGACCACCA		
Granzyme K	Forward	CGGGAAGCAACTGTTGAAAT	XM_423832	[19]
	Reverse	GAGTCTCCCTTGCAAGCATC		
MDA5	Forward	GGACGACCACGATCTCTGTGT	NM_001193638.1	[20]
	Reverse	CACCTGTCTGGTCTGCATGTTATC		
CXCLi1	Forward	AACTCCGATGCCAGTG	NM_205018.1	[21]
	Reverse	TTGGTGTCTGCCTTGT		
CXCLi2	Forward	CATCATGAAGCATTCCATCT	NM_205498.1	[21]
	Reverse	CTTCCAAGGGATCTTCATTT		
TGF-β3	Forward	TCTTTACATTGACTTCCGAC	NM_205454.1	[21]
	Reverse	TCCTCCCAACATAGTACAAG		
MX1	Forward	AAGCCTGAGCATGAGCAGAA	NM_204609.1	[22]
	Reverse	TCTCAGGCTGTCAACAAGATCAA		
OASL	Forward	AGATGTTGAAGCCGAAGTACCC	NM_205041.1	[22]
	Reverse	CTGAAGTCCTCCCTGCCTGT		
ISG12-2	Forward	TCAATGGGTGGCAAAGGAG	NM_001001296.5	[22]
	Reverse	TACAGGGAGAGCAAAGAAGAGAAGA		
IFIT5	Forward	CAGAATTTAATGCCGGCTATGC	XM_421662.4	[22]
	Reverse	TGCAAGTAAAGCCAAAAGATAAGTGT		
USP18	Forward	CAACGTGGGAAGAGGAGAAA	XM_416398.3	[22]
	Reverse	ACTTCATGAGCGGAGAAGGA		
IRF3/7	Forward	ACTGACCAGCCCAGGAACTCT	NM_205372.1	[22]
	Reverse	AAGGCTTTCCCAACCACAAA		
SST	Forward	GGTCCACGGTTATGGTGAAAG	NM_205336.1	[22]
	Reverse	GGTCAGAAATCACAACTCAAGCA		
KHSRP	Forward	CAGCGGGGAAATGATTAAGAAG	NM_204277.1	[22]
	Reverse	TTTGTGTGTGGGGATGGAGA		
Perforin	Forward	ATGGCGCAGGTGACAGTGA	XM_425355	[23]
	Reverse	TGGCCTGCACCGGTAATTC		
PARP	Forward	ATTGTGGAGGAGCTGGGAGGAA	NM_205263	[23]
	Reverse	AGGCTTGCTGCACTTCCCATC		
HMG-2	Forward	AGAGCACAAGAAGAAGCAC	M80574	[23]
	Reverse	GTCTTTTAGGAGCGTTGGGGTC		
NK lysin	Forward	GATGGTTCAGCTGCGTGGGATGC	DQ186291	[23]
	Reverse	CTGCCGGAGCTTCTTCAACA		

## Supplementary Materials

The following are available online at https://www.mdpi.com/1999-4915/12/1/114/s1.
