# Supplementary material for: Systematic Identification of Host Immune Key Factors Influencing Viral Infection in PBL of ALV-J Infected SPF Chicken
Source: Viruses. 2020 Jan 16;12(1):114. doi: 10.3390/v12010114 (PMC7019883; doi:10.3390/v12010114)
Supplement: Supplementary file 1 [file viruses-12-00114-s001.zip › supplementary file/Supplementary table 1.docx]

Supplementary table 1 Viremia and antibody level in four infected but antibody negative chicken

| Sample ID | 7DPI | 14DPI | 21DPI | 28DPI | 35DPI | 42DPI | 49DPI | 56DPI | 63DPI |
| --- | --- | --- | --- | --- | --- | --- | --- | --- | --- |
|  | Viremia antibody | Viremia antibody | Viremia antibody | Viremia antibody | Viremia antibody | Viremia antibody | Viremia antibody | Viremia antibody | Viremia antibody |
| #1 | 1.964 0.034 | 2.197 0.032 | 0.389 0.32 | 0.006 0.46 | — — | — — | — — | — — | — — |
| #2 | 1.989 0.02 | 1.92 0.04 | 0.009 0.44 | 0.006 0.47 | — — | — — | — — | — — | — — |
| #12 | 1.728 0.045 | 0.045 0.03 | 0.007 0.41 | 0.031 0.24 | 0.004 0.07 | 0.006 0.16 | 0.005 0.14 | 0.01 0.11 | 0.004 0.19 |
| #15 | 1.904 0.045 | 0.185 0.038 | 0.006 0.44 | 0.004 0.53 | 0.021 0.35 | 0.006 0.48 | 0.006 0.26 | 0.00 0.41 | — — |

Note: Chicken #1 and #2 were humanely sacrificed at 28 DPI. No sampling of #15 was detected at 63 DPI.
