# Supplementary figures and images for: Systematic Identification of Host Immune Key Factors Influencing Viral Infection in PBL of ALV-J Infected SPF Chicken
Source: Viruses. 2020 Jan 16;12(1):114. doi: 10.3390/v12010114 (PMC7019883; doi:10.3390/v12010114)

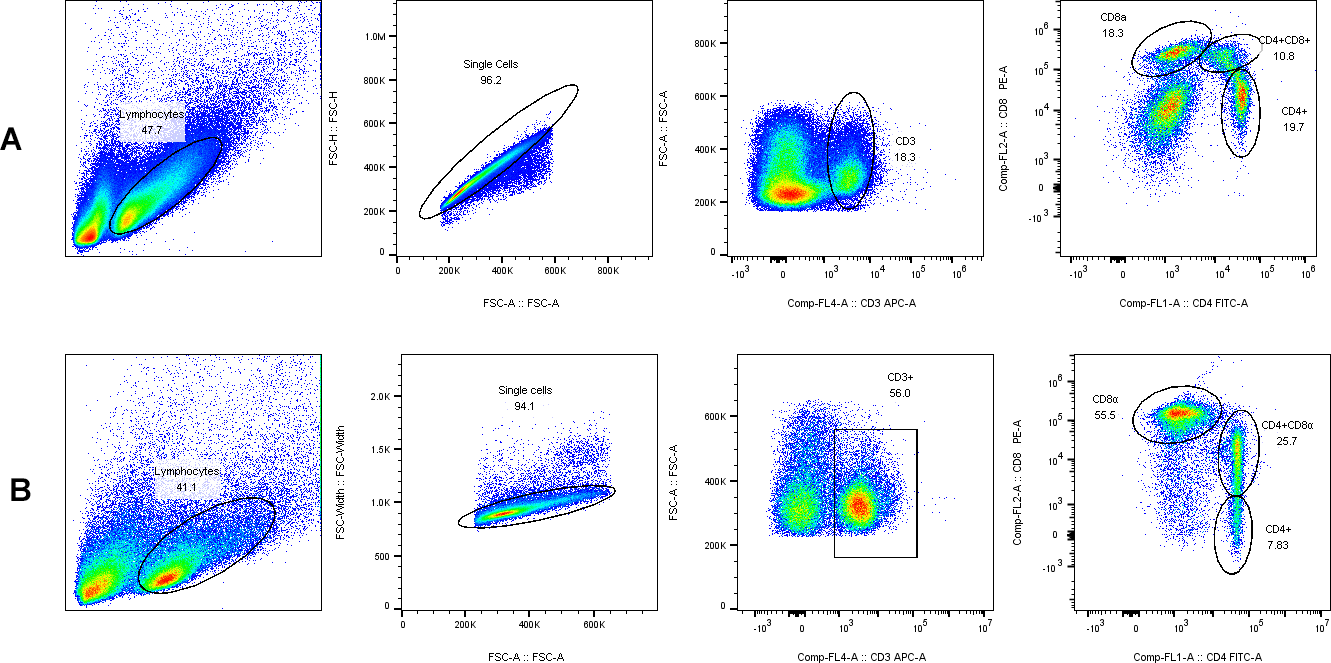

Supplement: Supplementary file 1 [file viruses-12-00114-s001.zip › supplementary file/Supplementary Fig1.tif]
